# Examination of CA1 Hippocampal DNA Methylation as a Mechanism for Closing of Estrogen’s Critical Window

**DOI:** 10.3389/fnagi.2021.717032

**Published:** 2021-08-04

**Authors:** Puja Sinha, Asha Rani, Ashok Kumar, Alberto Riva, Jason Orr Brant, Thomas C. Foster

**Affiliations:** ^1^Department of Neuroscience, McKnight Brain Institute, University of Florida, Gainesville, FL, United States; ^2^Bioinformatics Core, Interdisciplinary Center for Biotechnology Research, University of Florida, Gainesville, FL, United States; ^3^Department of Biostatistics, University of Florida, Gainesville, FL, United States; ^4^Genetics and Genomics Program, University of Florida, Gainesville, FL, United States

**Keywords:** estrogen, hippocampus, aging, DNA methylation, critical window, NMDA receptor

## Abstract

There is a critical window for estrogen replacement therapy, beyond which estradiol (E2) fails to enhance cognition and N-methyl-D-aspartate (NMDA) receptor function, and E2-responsive transcription decreases. Much less attention has been given to the mechanism for closing of the critical window, which is thought to involve the decline in estrogen signaling cascades, possibly involving epigenetic mechanisms, including DNA methylation. This study investigated changes in DNA methylation in region CA1 of the hippocampus of ovariectomized female rats over the course of brain aging and in response to E2-treatment, using whole genome bisulfite sequencing. Differential methylation of CpG and non-CpG (CHG and CHH) sites and associated genes were characterized in aged controls (AC), middle-age controls (MC), and young controls (YC) and differential methylation in response to E2-treatment (T) was examined in each age group (AT-AC, MT-MC, and YT-YC). Possible candidate genes for the closing of the critical window were defined as those that were hypomethylated by E2-treatment in younger animals, but were unresponsive in aged animals. Gene ontology categories for possible critical window genes were linked to response to hormones (*Adcyap1*, *Agtr2*, *Apob*, *Ahr*, *Andpro*, *Calm2*, *Cyp4a2*, *Htr1b*, *Nr3c2*, *Pitx2*, *Pth*, *Pdk4*, *Slc2a2*, *Tnc*, and *Wnt5a*), including G-protein receptor signaling (*Gpr22* and *Rgs4*). Other possible critical window genes were linked to glutamate synapses (*Nedd4*, *Grm1*, *Grm7*, and *Grin3a*). These results suggest that decreased E2 signaling with advanced age, and/or prolonged E2 deprivation, results in methylation of E2-responsive genes, including those involved in rapid E2 signaling, which may limit subsequent transcription.

## Introduction

Research suggests there is a critical window for estrogen replacement therapy (ERT) in providing neuroprotection and preserving cognition during aging. Delivery of estradiol (E2) is most effective in enhancing cognitive function when the treatment is initiated perimenopausally during middle-age, and ERT fails to promote neuroprotection and cognitive function if delivered several years after menopause in humans or months after a decline in circulating estrogen in rodents (Gibbs, 2000; Foster et al., 2003; Sherwin, 2009; Rocca et al., 2011; Daniel, 2013). Much of the work to understand E2 mechanisms of action have focused on the CA1 region of the hippocampus in rodent models. In younger animals, E2 acts to stimulate growth and the maintenance of dendritic spines in the CA1 region (Gould et al., 1990; Woolley et al., 1990) and the ability to modify CA1 spine plasticity declines with advanced age (Adams, Shah et al., 2001; Morrison et al., 2006). Moreover, E2 acts on membrane estrogen receptors to rapidly influence CA1 physiology in a manner opposite that observed during aging (Vouimba et al., 2000; Kumar and Foster, 2002; Foster et al., 2003, 2008; Foy et al., 2008; Witty et al., 2012). Rapid effects include activation of signaling pathways that regulate learning and memory (Packard, 1998; Frick and Kim, 2018). E2-mediated rapid effects on physiology and downstream signaling interact with genomic mechanisms to influence transcription of genes that decline with advancing age, including genes linked to transcriptional regulation, growth, synaptic activity, and neuroprotection (Foster, 2005; Bean et al., 2014). Finally, long-term (48–72 h post treatment) effects of E2 on memory are linked to an E2-induced increase in N-methyl-D-aspartate (NMDA) receptor mediated synaptic transmission and an inability of E2 treatment to increase NMDA receptor function is an indication of closing of the critical window (Woolley et al., 1997; Smith and McMahon, 2006; Snyder et al., 2011; Vedder et al., 2014; Bean et al., 2015).

Much less attention has been given to the mechanism for closing of the critical window, which is thought to involve the decline in estrogen receptor activation and an inability to initiate transcriptional processes normally observed in younger animals (Foster, 2005; Aenlle and Foster, 2010; Bean et al., 2014, 2015). One possible mechanism for the decline in E2-responsiveness involves epigenetic modifications, including DNA methylation. For DNA methylation, a methyl group is added or removed from cytosine nucleotides, particularly for cytosine nucleotides that are located next to a guanine nucleotide (i.e., CpG sites); although non-CpG sites (CHG and CHH) in brain are also sensitive to development, aging, and cognition (Numata et al., 2012; Lister et al., 2013; Guo et al., 2014; Ianov et al., 2017b). Environmental influences, including hormone status, act through DNA methylation to alter the availability of DNA to transcription, providing a potential link between environmental/lifestyle factors and the trajectory of age-related cognitive decline (Barter and Foster, 2018).

In the case of the critical window, altered signaling due to senescent physiology and decreased estrogen levels can render the genes normally activated by this signaling vulnerable to methylation changes (Bean et al., 2014; Ianov et al., 2017a; Barter and Foster, 2018). Thus, the present study was aimed at investigating DNA methylation in region CA1 of the hippocampus during brain aging and in response to E2-treatment, by analyzing the DNA methylome using whole genome bisulfite sequencing (WGBS). The results present several novel findings that relate to the idea that alterations in DNA methylation occur during aging and we provide evidence that closing of the critical window can be characterized by an inability to hypomethylate genes involved in the response to E2.

## Materials and Methods

### Animals

Fisher 344 female rats of three different age groups, young (5–6 months, *n* = 10), middle-age 10–12 months, *n* = 10), and aged (18–20 months, *n* = 11) were used in this study. The rats were housed two per cage and maintained under standard laboratory conditions with 12:12 light/dark cycles. All measures involving animal subjects were reviewed and approved by the Institutional Animal Care and Use Committee (IACUC) at the University of Florida (study protocol numbers: 201702226 and 20202226) and were executed in agreement with guiding principles established by the United States Public Health Service Policy on Humane Care and Use of Laboratory Animals. Food and water were provided *ad libitum* until ovariectomy (OVX), after which animals were switched to a casein-based chow (Cincinnati Lab Supply); this diet has lower levels of phytoestrogens compared with soy-based rat chow.

### Vaginal Lavages and Cytological Evaluation

The vaginal lavage and cytological evaluation were performed in the morning each day for 2–3 weeks before OVX and 1 week following OVX to confirm the removal of ovaries, as described previously (Bean et al., 2014). The procedure involves collecting vaginal secretions using a glass eye dropper with one drop of sterile 0.9% saline. Secretions were placed on a slide and the different phases of the estrous cycle were analyzed in wet conditions using a light microscope on low magnification. The cytological evaluation determines the cell type cornified, squamous, epithelial, leukocytes, and/or nucleated epithelial cells found in the different stages of the estrous cycle (proestrus, estrus, metestrus, diestrus) (McLean et al., 2012; Bean et al., 2015).

### Ovariectomy

Female rats ovarectomized bilaterally using aseptic procedures as described previously (Kumar and Foster, 2002; Sharrow et al., 2002; Foster et al., 2003; Bean et al., 2015). Briefly, isoflurane (Piramal Healthcare) was administered in O_2_ using an isoflurane anesthesia system (VetEquip). Induction of anesthesia was initiated within an induction chamber using a 4% concentration of isoflurane. After transfer to a nose cone, a 1.5–2% concentration was used for maintenance of the surgical plane of anesthesia. O_2_ was delivered at 1 L/min. The ovaries were removed and the overlying muscle was sutured and the skin stapled to close the incisions. Subcutaneous injections of buprenorphine (0.03 mg/kg) and saline (∼5 ml) were given for pain and hydration, respectively.

### Estrogen Treatment and Tissue Collection

Injections of 17β-estradiol-3-benzoate [E2 treated:10 μg, young (YT), *n* = 6; middle-age (MT), *n* = 6; aged (AT), *n* = 7] or oil vehicle [oil controls: young (YC), *n* = 4; middle-age (MC), *n* = 4; aged (AC), *n* = 4] were given subcutaneously at 6 weeks after OVX, for two consecutive days 24 h apart. The injections were delivered over two consecutive days, 24 h apart, in order to approximate the rodent estrous cycle. The dose of 10 μg has previously been shown to have effects on hippocampal-dependent cognition and CA1 spine density (Frick et al., 2004; Luine, 2014; Bean et al., 2015). Rats were euthanized 6 h after the last injection for tissue collection in order to be consistent with our previous work examining E2 effects on transcription (Aenlle et al., 2009; Aenlle and Foster, 2010; Han et al., 2013). *In vivo* studies indicate transcription peaks 1–2 h after treatment, with most studies showing increased mRNA levels at 6 h after treatment (Shull and Gorski, 1985; Cullinan-Bove and Koos, 1993; Hewitt et al., 2012). At the time of euthanasia, blood samples were collected and the CA1 region was dissected from dorsal hippocampi and flash frozen in liquid nitrogen, and stored at −80°C. Blood was centrifuged at 1,000 × *g* 4°C for 10 min to collect plasma and stored at −80°C for subsequent analysis. Plasma E2 levels were evaluated using a mouse/rat estradiol ELISA kit (Calbiotech). In addition, right uterine horn sections (cut at the base) of about 1-cm in length were removed and weighed to confirm efficacy of estrogen treatments.

### DNA Isolation

**FIGURE 1:**
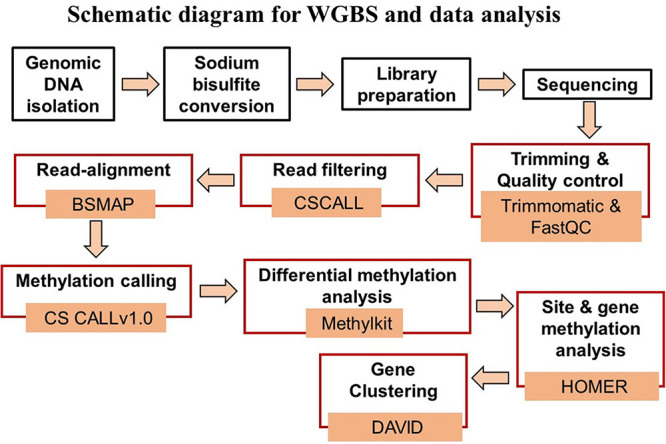
Schematic diagram of the steps for WGBS (black boxes) and data analysis (red boxes), and associated data analysis programs.

Figure 1 provides a schematic diagram of the pipeline for WGBS and data analysis. Genomic DNA was isolated from the hippocampal CA1 region for each animal. Tissue was weighed and washed with phosphate buffer saline (PBS) for homogenization in SET buffer [Sucrose 20% w/v (Sigma), 50 mM Tris–HCL pH 8.0 (Invitrogen), 50 mM EDTA pH 8.0 (Invitrogen)]. DNA extraction was done with the help of standard protocol involving SDS (Invitrogen) – Proteinase K (Millipore Sigma) treatment followed by phenol:chloroform:isoamyl alcohol (25:24:1, v/v, Thermo Fisher Scientific). The final DNA precipitation was carried out by chilled absolute ethanol and 3M sodium acetate (pH 5.2) (Sigma-Aldrich). The dissolved DNA was quantified by NanoDrop (A260/280 ratio greater than 1.80) (Thermo Fisher Scientific) and Qubit followed by visualization on 0.8% agarose gel (Thermo Fisher Scientific) for quality assessment.

### Genomic DNA Fragmentation and WGBS Library Preparation

Whole genome bisulfite sequencing libraries were prepared using two different library preparation kits due to product discontinuity. Libraries were made using TruSeq DNA Methylation kit (Illumina) and NEBNext^®^ Ultra^TM^ II DNA Library Prep Kit for Illumina^®^ (Cat No. E7645S) according to manufacturer’s instructions. For libraries prepared from NEB, in brief, genomic DNA 500 ng was used as input for fragmentation to generate 300 bp fragment size using Covaris S220. The fragmented samples were bead purified using Agencourt AMPure XP beads (1X) and Qubit quantified. Input mass of 100 ng-purified fragment was used for end preparation followed by ligation of methylated adaptor (NEBNext^®^ Multiplex Oligos for Illumina^®^ Cat No. E7535S) and uracil-specific excision reagent (USER) enzyme addition. Cleanup of adaptor ligated DNA was performed using the same Agencourt AMPure XP beads (0.85X) and quantified by Qubit dsDNA HS Assay. Sodium bisulfite conversion was done as instructed in the manual using the EpiMark Bisulfite Conversion kit (Cat No. E3318), and the bisulfite converted DNA was PCR amplified for 14 cycles using EpiMark Hot Start Taq DNA polymerase (Cat No. M0490) with the addition of a unique barcode (NEBNext^®^ Multiplex Oligos for Illumina^®^ Index Primers Set 1 and 3 Cat No. E7535/E7710) per library for multiplex sequencing. The resulting amplified libraries underwent two rounds of bead purification (0.8X) and were quantified with Qubit dsDNA HS Assay. Size distribution was analyzed in the TapeStation with the High Sensitivity D1000 Screen Tape. For libraries made with the Illumina TruSeq DNA Methylation kit, in brief, bisulfite converted DNA was annealed with the DNA synthesis primer followed by the synthesis of DNA containing a specific sequence tag from the random hexamers. Tagging of the synthesized DNA and purification with beads was performed for optimal size selection. Library was selected based on double-sided method for obtaining absolute size range. The ratio 0.75 was used as left side and the right size ratio used was 0.64, resulting in the ratio difference of 0.11 (left side minus right side ratios). Following size selection, amplification of the libraries was performed with a total of 12 cycles and the addition of a unique barcode per library for multiplex sequencing was done with the Index PCR Primers. Finally, libraries were purified using the Agencourt AMPure XP beads and quantified by using Qubit dsDNA HS Assay. Size distribution was analyzed in the TapeStation with the High Sensitivity D1000 Screen Tape.

### Sequencing

Libraries were paired-end sequenced for 150 bp with the Illumina HiSeq3000 (2 × 150 cycles) at the University of Florida Interdisciplinary Center for Biotechnology Research (ICBR) core, as described previously (Ianov et al., 2017a, b). Multiplex sequencing was performed with RNA-seq libraries provided by the ICBR core to introduce base diversity and efficient base call generation. In addition, 1% of PhiX spike-in control was added to improve sequencing quality. On average, 74% of the resulting reads per biological samples were uniquely mapped to the rat reference genome (rn6). The data for this study has been uploaded to NCBI’s Gene Expression Omnibus under the accession number: GSE167038.

### Bio-Informatics and Statistical Data Analysis

Data analysis was performed using the differential methylation analysis pipeline (DMAP2) available at the University of Florida high performance computer (HPC) clusters (Figure 1). DMAP2 is a modified and efficient pipeline based on Model based Analysis of Bisulfite Sequencing data (MOABS) for the analysis of large-scale single base-resolution DNA methylation data. In brief, the pipeline involves the reading of input data sets in a FastQ format followed by short reads trimming using the program trimmomatic (Bolger et al., 2014) and quality control by FastQC and multiQC, which summarizes the analyzed results for multiple tools and samples in a single report (Ewels et al., 2016). The read filtering was done using CSCALL to remove reads containing more than 10,000 or more than 5.0% “lone” cytosines (defined as cytosines that are not part in one of the following sites: CG and GC). The input reads were aligned to the rn6 reference genome using BSMAP (Xi and Li, 2009). Methylation calling was performed with CSCALL v1.0 (Riva, 2016) using the rn6-CG index. MethylKit package was used for differential methylation analysis considering the two library preparation kits as covariates to minimize its influence on methylation statistics (Akalin et al., 2012). Differentially methylated sites for which the *p*-value of the difference between test and control methylation rates was below 0.01 were considered significant. Gene annotation was performed using HOMER with annotate peaks.pl, which provides detailed annotation for the number of differentially methylated sites present in specific region including promoters (−1,000 bp to +1,000 bp) and gene body regions (Heinz et al., 2010).

For the analysis of gene enrichment and functional annotation clustering, both hypermethylated or hypomethylated datasets for genes, identified by HOMER, were submitted separately to the NIH database for annotation, visualization and integrated discovery (DAVID, version 6.8) (Huang et al., 2007a, b). Age differences resulted in a large number of differentially methylated genes. Therefore, non-directed cluster analysis was employed and cutoff selection was based on a Benjamini False Discovery Rate (FDR) *p* < 0.05 and “FAT” categories were used for gene ontology (GO) annotation, UniProtKB keyword, and Kyoto Encyclopedia of Genes and Genomes (KEGG) pathways. Hypothesis testing for a subset of specific clustered was performed with a cutoff of *p* < 0.05.

### Transcription Factor Binding Motif Enrichment Analyses

For determining the binding sites of certain transcription factors (TFs), the “findMotifsGenome.pl” script in the motif analysis program HOMER (Heinz et al., 2010) was used with default settings (Masked, Region Size 200 bp, Motif length 8, 10, 12) to test for enrichment of known/*de novo* vertebrate binding motifs. Differentially methylated regions (DMRs) of E2-treated relative to oil-matched controls from young, middle age, and aged cohorts obtained from the MethylKit package were used to find enriched motifs. Results from the top 10 motif enrichment analyses for young, middle-age, and aged E2-treatment groups were provided in section “Results.”

## Results

### CpG Methylation During Aging in OVX Female Control Animals

The results for the vaginal lavage and cytological evaluation prior to surgery indicated that young and middle-aged rats were cycling at regular intervals of ∼5 days and aged animals exhibited an irregular estrus cycle or prolonged diestrus. E2 treatment increased uterine weight [*t*(33) = 6.91, *p* < 0.005; E2 = 0.16 ± 0.02 g mean ± SEM, oil = 0.07 ± 0.004]. Plasma E2 levels were measured for a subset of animals (E2 treated = 12, oil treated = 5). A *t*-test indicated a significant [*t*(15) = 3.75, *p* < 0.005; E2 = 160.9 ± 26.9 pg/ml mean ± SEM, oil = 1.6 ± 0.4] elevation of E2 due to treatment.

**FIGURE 2:**
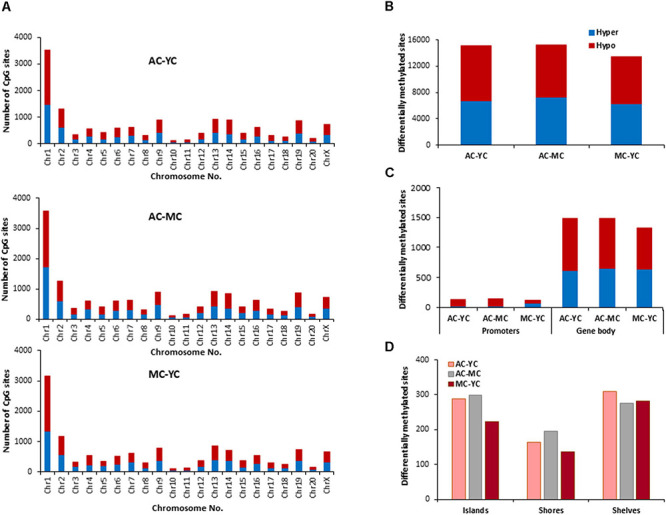
Age-related differences in DNA methylation at CpG sites of control animals. **(A)** Differentially methylated CpG sites along the chromosome (1–20) including X-chromosome in control groups. The differential methylation was based on a statistical significance of *p* < 0.01. **(B)** Total number of differentially methylated CpG sites across the control groups. The red bars denote hypomethylation and blue bar denotes hypermethylation for the older group relative to the younger group. **(C)** Age differences in the number of differentially methylated CpG sites in promoter and gene body regions. **(D)** Comparison of the differentially methylated CpG sites across the regulatory regions: islands, shores, and shelves.

The total number of CpG sites across all groups was ∼2.5 million. Age differences in CpG methylation between the control groups for all ages are illustrated in Figures 2A–D. The total number of differentially (*p* < 0.01) hyper- and hypomethylated CpG sites was greater in the oldest animals (Figure 2B). Thus, compared to the YC group, AC animals exhibited 15,104 total differentially methylated sites (6,746 hypermethylated and 8,358 hypomethylated). When the AC group was compared to MC animals (AC-MC), the number of total differentially methylated sites was 15,237 (7,257 hypermethylated and 7,980 hypomethylated) for aged animals. There were fewer differences when comparing MC and YC (MC-YC), with 13,486 sites that were differentially methylated (6,318 hypermethylated and 7,168 hypomethylated). The same pattern was evident for promoter and gene body regions (Figure 2C), and comparable for islands, shores, and shelves (Figure 2D), such that the number of differentially methylated CpG sites was greater for AC when compared to the other two groups and minimal difference were observed for the MC-YC comparison. In general, older animals exhibited more sites that were hypermethylated (>50%) compared to MC and YC, while the MC-YC comparison indicated that MC exhibited more hypomethylation (>50%) relative to YC.

The genes linked to differential hypermethylated and hypomethylated CpG sites, across age, were identified by HOMER (Supplementary Table 1) and submitted to DAVID for analysis of cluster enrichment. The top 10 pathways and associated genes for each comparison are included in Supplementary Table 1. In many cases, the same gene exhibited both hypermethylated and hypomethylation. A common theme across all comparisons was clustering for GO biological processes and cellular structures related to neuronal growth/development and the synapse, such that the same GO categories were observed for hypermethylated and hypomethylated genes. Absent from the list were GO terms linked to the immune system. A few categories (behavior, steroid hormone response, and GABA receptor complex) exhibited relatively age-specific change in methylation. The number of differentially methylated sites was lowest between MC-YC (Figure 2) and for this comparison, 1,173 hypomethylated and 1,199 hypermethylated genes were in the DAVID database. The MC group exhibited hypermethylation of genes that clustered for behavior and response to steroid hormone. Compared to the MC or YC groups, the AC group exhibited more robust changes in hypomethylated and hypermethylated genes. For the AC-YC comparison, aged animals exhibited 1,269 hypomethylated genes linked to the response to steroid hormone. Enrichment of 1,199 hypermethylated genes was observed for GO terms related to the GABA receptor complex. Similarly, for the AC-MC comparison, older animals continued to exhibit clustering for hypomethylated genes (1,226) linked to behavior and response to steroid hormone. The 1,217 hypermethylated genes, for the AC-MC comparison, exhibited clustering for the GABA receptor complex.

### Effect of E2 on CpG Methylation During Aging

**FIGURE 3:**
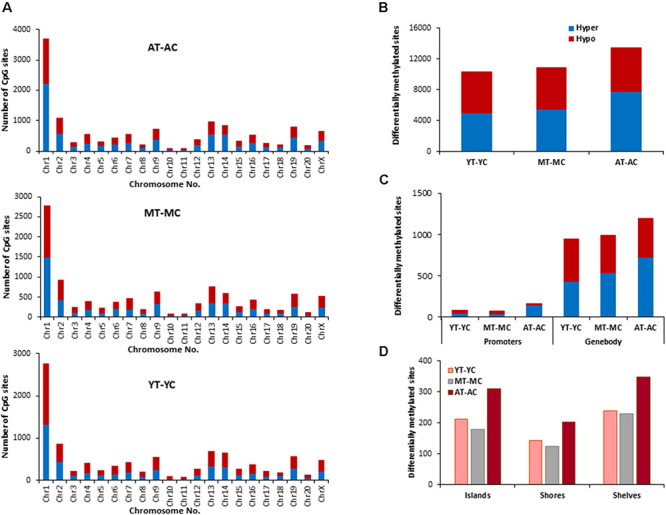
Age-related differences in DNA CpG methylation due to E2-treatment. **(A)** Differentially methylated CpG sites along the chromosome (1–20) including X-chromosome for each age. The differential methylation was based on a statistical significance of *p* < 0.01. **(B)** Total number of differentially methylated CpG sites induced by E2-treatment for each age group. The red bars denote hypomethylation and blue bar denotes hypermethylation for the E2-treatment group relative to the age-matched control group. **(C)** Number of differentially methylated CpG sites in promoter and gene body regions in E2-treated relative to age-matched controls. **(D)** Comparison of the differentially methylated CpG sites across the regulatory regions: islands, shores, and shelves.

The distribution of E2 induced methylation changes associated with each age was similar across chromosomes. In general, a higher number of differentially methylated CpG sites was found on chromosomes 1 in all groups (Figure 3A). E2-treatment had the greatest and least effect in older and younger groups, respectively. Thus, the total number of differentially (*p* < 0.01) methylated CpG sites between control and treatment groups increased with age from 10,270 for YT-YC, to 10,913 for MT-MC, and 13,486 for AT-AC (Figure 3B). For young animals, E2-treatment was associated with more hypomethylated sites and aged animals exhibited an increase proportion of hypermethylated sites, such that the percent hypermethylation relative to total was 48% in YT-YC and 58% in AT-AC. The pattern of E2-mediated differences was also evident in the promoter and gene body regions (Figure 3C), such that the total number of differentially methylated sites in these regions increased with increasing age. The age-related increase in the number of modified sites was also evident for islands, shores, and shelves (Figure 3D), which exhibited an approximately 1.5-fold increase in the number of differentially methylated sites for aged animals relative to the other two groups.

The identified CpG sites, were analyzed for differentially methylated genes associated with E2-treatment, which were annotated by HOMER (Supplementary Table 1) and cluster analysis was performed with DAVID. The top 10 pathways and associated genes for each comparison are included in Supplementary Table 1. Despite the apparent difference in the number of differentially methylated sites, the number of differentially methylated genes was similar across age groups. Again, hypermethylated and hypomethylated sites were enriched for neuronal and synaptic genes. For young animals (YT-YC), E2-treatment resulted in hypomethylation of 849 genes and hypermethylation of 816 genes. Enrichment clusters were similar for hypomethylated and hypermethylated genes, with the possible exception of hypermethylation of genes involved in response to lipids and positive regulation of cation transmembrane transport. For middle-age animals (MT-MC), E2-treatment resulted in hypomethylation of 876 genes and hypermethylation of 851 genes. Again, cluster analysis indicated similar categories for hypomethylated and hypermethylated genes, with a few GO terms specific for hypomethylated (response to organic cyclic compound and calcium ion binding) and hypermethylated genes (chemotaxis and axon guidance). For aged animals (AT-AC), E2-treatment resulted in hypomethylation of 820 genes and hypermethylation of 878 genes, with a number of common categories linked to the synapse and nervous system development. Cluster analysis indicated hypomethylation was also linked to genes for regulation of synapse structure or activity, negative chemotaxis, and cell morphogenesis involved in differentiation. Hypermethylation was linked to genes for somatodendritic compartment and neurogenesis.

In other systems, a decrease in estrogen signaling promotes DNA methylation of E2-responsive genes (Leu et al., 2004; Stone et al., 2015; Levine et al., 2016). Furthermore, closing of the critical window for E2-beneficial effects is associated with an inability of E2-treatment to induce transcription of E2-responsive genes. Therefore, we hypothesized that decreased E2-responsiveness with age is due to an inability to remove methyl groups, in order to induce transcription. Therefore, genes that exhibited CpG hypomethylation associated with E2-treatment in both young and middle-age animals (YT-YC and MT-YC) were identified. This resulted in 525 genes that exhibited CpG hypomethylation in both YT and MT, relative to age-matched controls. For the AT-AC comparison, 135 of the 525 genes did not exhibit hypomethylation and were considered non-responsive to E2-treatment. Non-directed cluster analysis indicated regulation of nervous system development (GO:0007399, 31 genes, adj. *p*-value 0.042), cell division (GO:0051302, eight genes, adj. *p*-value 0.026), establishment or maintenance of cell polarity (GO:0007163, eight genes, adj. *p*-value 0.042), response to organic substance (GO:0010033, 41 genes, adj. *p*-value 0.0094), response to oxygen-containing compound (GO:1901700, 27 genes, adj. *p*-value 0.042), and response to endogenous stimulus (GO:0009719, 26 genes, adj. *p*-value 0.049). Analysis directed at response to hormone was significant (GO:0009725, 17 genes, *p* = 0.0012; *Adcyap1*, *Agtr2*, *Apob*, *Ahr*, *Andpro*, *Calm2*, *Cyp4a2*, *Gpr22*, *Htr1b*, *Nedd4*, *Nr3c2*, *Pitx2*, *Pth*, *Pdk4*, *Slc2a2*, *Tnc*, and *Wnt5a*) and nine of these non-responsive genes are linked to the GO term response to steroid hormone (GO:0048545, 9 genes, *p* = 0.019).

### Non-CpG Methylation During Aging in OVX Female Control Animals

#### Genes Linked to CHG Sites

**FIGURE 4:**
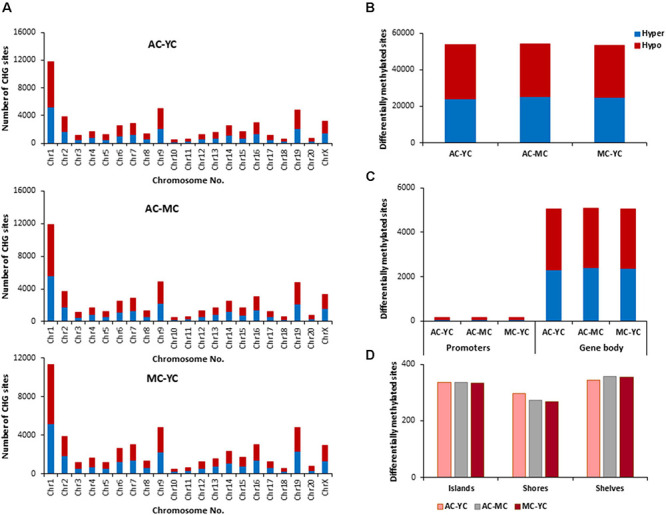
Age-related differences in DNA methylation at CHG sites of control animals. **(A)** Differentially methylated CHG sites along the chromosome (1–20) including X-chromosome in control groups. The differential methylation was based on a statistical significance of *p* < 0.01. **(B)** Total number of differentially methylated CHG sites across the control groups. The red bars denote hypomethylation and blue bar denotes hypermethylation for the older group relative to the younger group. **(C)** Age differences in the number of differentially methylated CHG sites in promoter and gene body regions. **(D)** Comparison of the differentially methylated CHG sites across the regulatory regions: islands, shores, and shelves.

The total number of CHG sites across all groups was ∼12 million. The number of age-related differentially (*p* < 0.01) methylated CHG sites increased relative to CpG sites and the number of differentially methylated sites was similar across age comparisons (Figure 4). The genes identified by HOMER and linked to differential methylation of CHG sites across age (Supplementary Table 2), were submitted to DAVID for analysis of cluster enrichment. The top 10 pathways and associated genes for each comparison are included in Supplementary Table 2. Similar to CpG sites, across all age groups, hypomethylated and hypermethylated genes clustered for similar GO terms related to neuronal development and the synapse. Comparison of MC-YC differences indicated hypomethylation of 2,025 CHG genes and hypermethylation for 2,035 CHG genes. For enrichment clusters that exhibited methylation state differences, MC hypomethylation was observed for pathways linked to cation homeostasis and oxidative stress, and MC hypermethylation was observed for GO terms linked to the GABA-A receptor complex, stress-activated MAPK pathway, and the UniProtKB Keyword adaptive immunity. Cluster analysis of CHG sites for the AC-YC comparison indicated hypomethylation of 2,027 genes and hypermethylation of 1,982 genes. Differential methylation was observed as AC hypomethylation for pathways linked to response to nitrogen compound and oxidative stress, and AC hypermethylation was observed for cation homeostasis and stress-activated MAPK pathway. Cluster analysis of CHG sites between AC-MC indicated hypomethylation of 2,057 genes and hypermethylation of 1,940 genes. AC hypomethylation was observed specifically for response to stress pathways, stress-activated MAPK pathway, and behavior. The AC group exhibited hypermethylation for the cation homeostasis and adaptive immunity. Thus, relative to the other two groups, the oldest group exhibited increased methylation for genes linked to cation homeostasis, middle-age groups exhibited increased methylation for genes linked to the stress-activated MAPK pathway and the young group exhibited hypomethylation of genes for oxidative stress. Hypermethylation was observed for adaptive immunity genes between MC-YC and AC-MC.

#### Genes Linked to E2-Responsive CHG Sites

**FIGURE 5:**
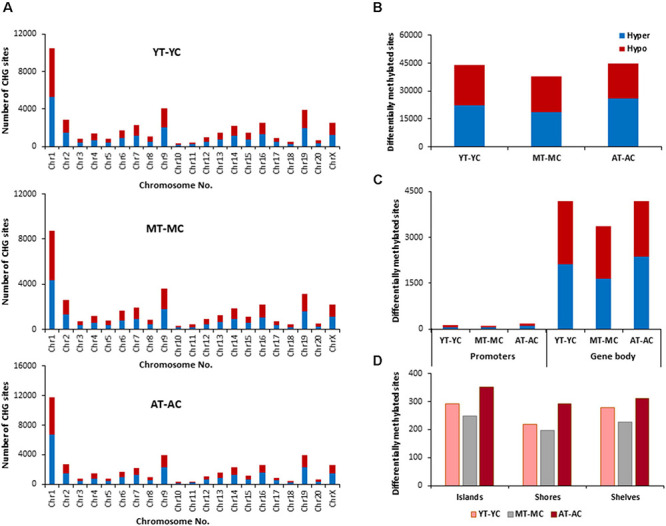
Age-related differences in DNA CHG methylation due to E2-treatment. **(A)** Differentially methylated CHG sites along the chromosome (1–20) including X-chromosome for each age. The differential methylation was based on a statistical significance of *p* < 0.01. **(B)** Total number of differentially methylated CHG sites induced by E2-treatment for each age group. The red bars denote hypomethylation and blue bar denotes hypermethylation for the E2-treatment group relative to the age-matched control group. **(C)** Number of differentially methylated CHG sites in promoter and gene body regions in E2-treated relative to age-matched controls. **(D)** Comparison of the differentially methylated CHG sites across the regulatory regions: islands, shores, and shelves.

The E2 associated pattern for differential (*p* < 0.01) methylation for CHG sites was somewhat different from the pattern for CpG sites. E2-treatment influenced more CHG sites in aged and young, relative to middle-age (Figures 5A–D). Again, the highest number of differentially methylated CHG sites was found on chromosomes 1 in all groups (Figure 5A) and aged animals appear to exhibit more responsive sites for islands, shores, and shelves (Figure 5D). The identified CHG sites were analyzed for differentially methylated genes (Supplementary Table 2), which were annotated by HOMER and mapped with DAVID. The top 10 pathways and associated genes for each comparison are included in Supplementary Table 2. Again, many of the same genes were hypermethylated and hypomethylated and enrichment was observed for similar GO terms linked to neurodevelopment and the synapse. Interestingly, the number of differentially methylated genes, following E2 treatment, was greatest for young (YT-YC, 3,194 genes) and lowest for aged animals (AT-AC, 2,735 genes). For young animals (YT-YC), E2-treatment resulted in CHG hypomethylation of 1,577 genes and hypermethylation of 1,617 genes. Differential enrichment was observed for E2-treatment hypomethylated genes linked to behavior, cation homeostasis, and GABAergic synapses. For middle-age animals (MT-MC), E2-treatment resulted in CHG hypomethylation of 1,534 genes and hypermethylation of 1,503 genes. Differential enrichment was observed for hypomethylated genes linked to a number of stress-related pathways including the stress-activated MAPK pathway and regulation of cellular response to oxidative stress. For aged animals (AT-AC), E2-treatment resulted in CHG hypomethylation of 1,336 genes and hypermethylation of 1,399 genes. Differential enrichment was observed for hypomethylated genes linked to the GABA-A receptor complex and hypermethylation was observed for positive regulation of stress-activated MAPK cascade.

To determined possible CHG genes linked to the decrease E2-responsiveness with age, genes were filtered to obtain genes exhibiting CHG hypomethylation associated with E2-treatment in both young and middle-age animals (YT-YC and MT-YC). This resulted in 1,213 genes that exhibited CHG hypomethylation in both YT and MT, relative to age-matched controls. For the AT-AC comparison, 222 of the 1,213 genes did not exhibit hypomethylation (i.e., non-responsive genes). Non-directed cluster analysis did not identify a significant GO biological process cluster. However, analysis directed at response to hormone was significant (GO:0009725, 25 genes, *p* = 0.00034; *Adra1a*, *Ahr*, *Andpro*, *Aqp4*, *Asip*, *Cckar*, *Cd55*, *Cyp4a2*, *Drd1*, *Erbb4*, *Esr1*, *Fbxo32*, *Fer*, *Gabrb1*, *Gpr22*, *Lnpep*, *Med30*, *Pfkfb1*, *Phex*, *Pik3r1*, *Ptgdr*, *Pth*, *Reg3b*, *Slc26a5*, and *Sts*) and 12 of these non-responsive genes are also linked to response to steroid hormone (GO:0048545, 12 genes, *p* = 0.022).

#### Genes Linked to CHH Sites

**FIGURE 6:**
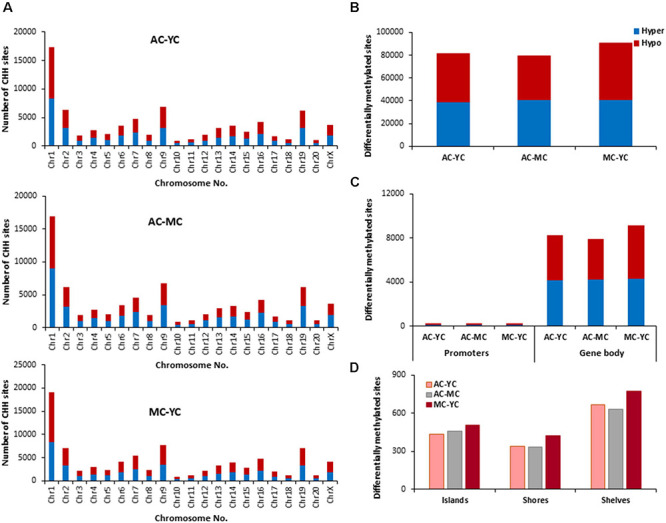
Age-related differences in DNA methylation at CHH sites of control animals. **(A)** Differentially methylated CHH sites along the chromosome (1–20) including X-chromosome. The differential methylation was based on a statistical significance of *p* < 0.01. **(B)** Total number of differentially methylated CHH sites across the control groups. The red bars denote hypomethylation and blue bar denotes hypermethylation for the older group relative to the younger group. **(C)** Age differences in the number of differentially methylated CHH sites in promoter and gene body regions. **(D)** Comparison of the differentially methylated CHH sites across the regulatory regions: islands, shores, and shelves.

The total number of CHH sites across all groups was ∼35 million. The number of age-related differentially (*p* < 0.01) methylated CHH sites (Figure 6) was increased ∼5 fold relative to CpG sites. The genes linked to differential methylation of CHH sites, across age (Supplementary Table 3), were submitted to DAVID for analysis of cluster enrichment. The top 10 pathways and associated genes for each comparison are included in Supplementary Table 3. As with CpG and CGH sites, hypomethylated and hypermethylated genes were similarly clustered for GO terms linked to neurodevelopment and the synapse. For the MC-YC comparison, HOMER identified 2,367 hypomethylated CHH genes and 2,342 hypermethylated CHH genes. Differential enrichment was observed for hypermethylated genes linked to ion homeostasis, including cation homeostasis and calcium ion homeostasis. For comparison of AC-YC groups, 2,249 genes were hypomethylated and 2,289 genes were hypermethylated. Again, hypermethylated CHH sites clustered for cation homeostasis. For hypomethylated genes, differential cluster enrichment was observed for genes linked to ligand-gated ion channel activity. For the AC-MC comparison, 2,173 genes were hypomethylated and differential enrichment was observed for genes linked to ligand-gated channel activity. For the 2,250 AC-MC hypermethylated genes, differential clustering was observed for genes linked to ion homeostasis and adaptive immunity. Thus, for oldest animals, differential methylation was observed with hypermethylated genes linked to ion homeostasis or adaptive immunity and hypomethylation of genes linked to ligand-gated channel activity.

#### Genes Linked to E2-Responsive CHH Sites

**FIGURE 7:**
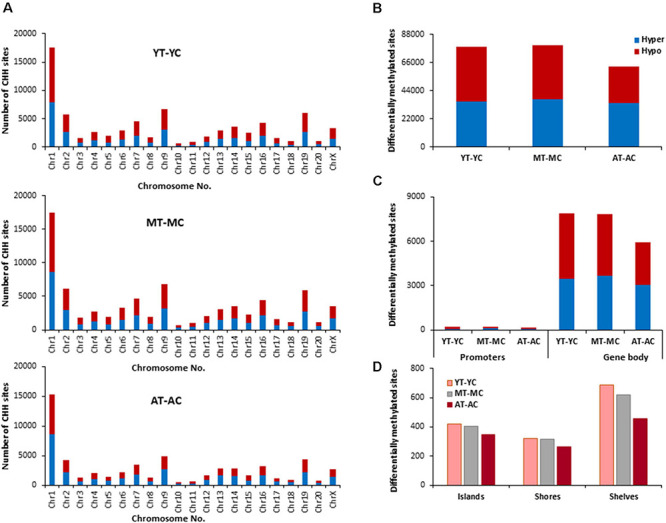
Age-related differences in DNA CHH methylation due to E2-treatment. **(A)** Differentially methylated CHH sites along the chromosome (1–20) including X-chromosome for each age. The differential methylation was based on a statistical significance of *p* < 0.01. **(B)** Total number of differentially methylated CHH sites induced by E2-treatment for each age group. The red bars denote hypomethylation and blue bar denotes hypermethylation for the E2-treatment group relative to the age-matched control group. **(C)** Number of differentially methylated CHH sites in promoter and gene body regions in E2-treated relative to age-matched controls. **(D)** Comparison of the differentially methylated CHH sites across the regulatory regions: islands, shores, and shelves.

The identified CHH sites that exhibited differential methylation from control and E2-treatment were analyzed for differentially methylated genes (Supplementary Table 3), which were annotated and mapped with DAVID. The top 10 pathways and associated genes for each comparison are included in Supplementary Table 3. Again, chromosome 1 was the most responsive (Figure 7). However, in contrast to CpG and CHG sites, aged animals appear to be less E2-responsive, exhibiting the lowest number of total sites, including those for islands, shores and shelves. Furthermore, the number of E2-responsive genes was decreased with aged advanced age (YT-YC 3,753 genes, MT-MC 3,799 genes, and AT-AC 3,061 genes). For each age group, E2-treatment resulted in hypermethylation and hypomethylation of many of the same genes resulting in clustering for many of the same GO terms. For young animals, 1,929 genes exhibited hypomethylation and 1,824 hypermethylated genes. For differentially methylated clusters, YT animals exhibited hypomethylation genes for voltage-gated potassium channel activity. For the hypermethylated genes, differential clustering was linked to behavior and oxidative stress. Middle-age animals exhibited 1,927 hypomethylated and 1,872 hypermethylated genes. Hypermethylated genes were associated with clusters for cellular response to hormone stimulus, including response to steroid hormone. For aged animals, 1,541 genes were hypomethylated and 1,520 were hypermethylated following E2-treatment. Differential methylation was observed for hypomethylated genes which were enriched for behavior and calcium ion binding.

To determined possible CHH genes linked to the decrease E2-responsiveness with age, genes were filtered to obtain a list of genes exhibiting CHH hypomethylation associated with E2-treatment in both young and middle-age animals. This resulted in 1,643 genes that exhibited CHH hypomethylation in both YT and MT, relative to age-matched controls (YT-YC and MT-YC). For the AT-AC comparison, 307 of the 1,643 genes did not exhibit hypomethylation and were considered possible markers of the critical window. Non-directed cluster analysis did not indicate a significant cluster for biological processes. Similarly, analysis directed at response to hormone was not significant (GO:0009725, 20 genes, *p* = 0.19); however, steroid hormone receptor binding was (GO:0035258, 6 genes, *p* = 0.0052; *Grm1*, *Isl1*, *Kdm4c*, *Ncoa1*, *Nr4a3*, and *Ppargc1a*).

### Enrichment of Transcription Factors Binding Sites

**FIGURE 8:**
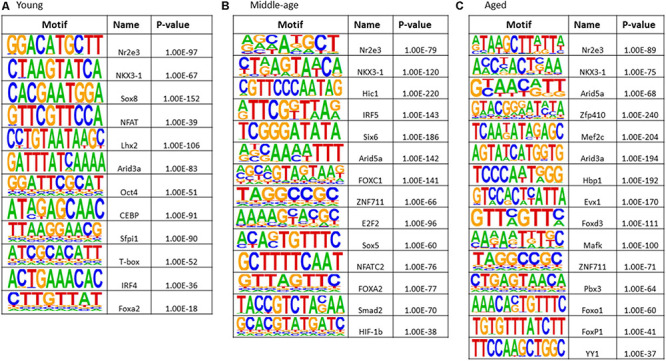
Transcription factor binding motif enrichment analyses for E2-treatment versus control in **(A)** young, **(B)** middle-age, and **(C)** aged animals.

The results of transcription factor motif enrichment, comparing treated versus control groups for each age, are shown in Figure 8. Common motifs across all age groups were linked to nuclear receptor activity (Nr2e3), estrogen receptor binding activity (Nkx3-1), and transcription regulation by RNA Polymerase II (Sox18). The young group exhibited enriched motifs Sox8 (nervous system development), Lhx2 (cell differentiation), and NFAT (nuclear factor of activated T-cells). Middle-age included FOXC1 (involved in cell surface receptor signaling pathway), Sox5 (neurogenesis), and hypoxia inducible factor-1b (HIF-1b). Motifs observed for middle-age and aged animals included Arid5a (role in estrogen receptor-mediated transcriptional regulation) and ZNF711 (neuron development). For aged animals, unique transcription factors (Mef2c, Hbp1, Evx1, Mafk, Pbx3, Foxo1, FOXP1, and YY1) were linked to nervous system development, ion transport, neuronal specification, oxidative stress, and cell cycle control.

## Discussion

The current study confirms a number of findings concerning age-related changes in DNA methylation and presents several novel results that relate to DNA methylation as an epigenetic marker for the closing of the critical window for E2 effects on the CA1 region of the hippocampus. Consistent with previous work in the whole hippocampus of mice, ∼0.5% of the potential methylation sites exhibit a changes in the hippocampus with age (Masser et al., 2017; Hadad et al., 2018). The number of differentially methylated CpG sites was minimized between YC and MC groups, relative to the AC group, for promoters and gene body regions and regulatory regions (islands, shores, and shelves). This finding is consistent with previous work demonstrating little change in methylation status between young adult to middle-age, and increased individual variability in methylation with advanced age (Numata et al., 2012; Lister et al., 2013).

Differential methylation associated with age was enriched for GO categories previously reported for studies examining methylation during aging, including nervous system development and the synapse (Horvath et al., 2012; Lister et al., 2013; Stubbs et al., 2017). In the current study, many of the same GO terms exhibited clustering for both hypermethylated and hypomethylated genes. Furthermore, due to multiple sites, the same gene can exhibit both hypermethylation and hypomethylation. Some specificity was observed with increased hypermethylation of CpG sites from YC to AC and CHG sites between MC-YC for genes linked to the GABA receptor complex. In addition, relative to young controls, the middle-age and aged control animals exhibited CHG hypomethylation of oxidative stress genes, and aged animals exhibited CHG and CHH hypermethylation of genes linked to cation homeostasis and immune processes, which are hallmarks of aging (Mattson and Arumugam, 2018; Foster, 2019; Mayne et al., 2020). The results provide support for the idea that non-CpG hypermethylation of immune response genes are characteristic of brain aging (Lee et al., 2020). However, the cause and effect relationship between DNA methylation and processes of aging remains to be defined (Konar et al., 2016; Berridge, 2017; Barter and Foster, 2018).

Closing of the critical window involves a decline in the ability to activate E2-responsive genes (Foster, 2005; Aenlle and Foster, 2010; Bean et al., 2014, 2015). The decline in E2-responsive transcription could be due to changes in E2-signaling from the receptor to the nucleus or to changes in DNA accessibility (Foster, 2005; Han et al., 2013; Bean et al., 2014, 2015; Barter and Foster, 2018). DNA methylation recruits suppressor complexes, hindering DNA-binding factors, and methylation of promoters, the gene body region, and intergenic regions is associated with decreased gene expression (Moore et al., 2013; Imamura et al., 2014; Kinde et al., 2016; Rajarajan et al., 2016; Barter and Foster, 2018; Jeong et al., 2021). In contrast, hypomethylation is associated with an increased ability to initiate transcription. Thus, genes that were hypomethylated by E2-treatment in young and middle-age animals and unresponsive in older animals were considered possible candidate markers or mechanisms for the closing of the critical window. Previous work indicates that genes linked to the GO term response to hormone, are sensitive to E2-treatment in the hippocampus of middle-age animals (Sarvari et al., 2015) and the current study found that potential critical window genes were also linked to the response to steroid hormones (CpG and CHG) and steroid hormone binding (CHH).

Hormone response genes, *Ahr*, *Andpro*, *Cyp4a2*, and *Gpr22*, were non-responsive for CpG and CHG sites of aged animals. Recent work suggests a link between estrogen signaling/deprivation, aging, memory, and *Ahr* expression (Bravo-Ferrer et al., 2019; Zhang et al., 2021) and the G-protein coupled receptor, *Gpr22*, has been linked to estrogen signaling involved in cardioprotection (Wang et al., 2018). Several genes were non-responsive in older animals across CpG, CHG, and CHH sites (*Andpro*, *Bcas1*, *Cyp4a2*, *Dpy19l4*, *Grin3a*, *Mbd2*, *Nalcn*, *Pth*, and *Rgs4*). Some of the non-responsive genes may directly contribute to the rapidly activated G-protein signaling cascades. For example, *Rgs4* is involved in G-protein receptor signaling and expression in the hippocampus is differentially sensitive to E2-treatment during aging (Aenlle and Foster, 2010; Sarvari et al., 2017). Other genes may be downstream of rapid signal transduction cascades that influence transcription (Foster, 2005; Bean et al., 2014). Indeed, differential enrichment of transcription factor motifs suggests the involvement of cofactors that regulate transcriptional activation or repression depending on the cell type or environmental context. These cofactors may determine E2 regulation in the cortex and muscle, respectively, of *Bcas1*, required for myelination, and the sodium leak channel, *Nalcn* (Humphreys et al., 2014; Amazu et al., 2020). Interestingly, treatment induced CpG hypomethylation of hormone response genes, limited to young and middle-age animals, mainly occurred at intergenic regions or within introns (Table 1). Methylation within introns may signal transcription of different splice variants. Intergenic regions can act as distal regulatory sites for gene expression and hypomethylation is associated with transcription factor binding and increased gene expression during development and maturation (Stadler et al., 2011; Sharma et al., 2016; Song et al., 2019; He et al., 2020). Methylation of intergenic regions is sensitive to hormone status in E2-responsive tissue (Houshdaran et al., 2020). In the brain, methylation of intergenic region exhibits large sex differences (Nugent et al., 2015; Masser et al., 2017) and can be observed in association with neurological disorders (Xiao et al., 2014; Sugawara et al., 2018; Aberg et al., 2020).

**TABLE 1:** DNA structure exhibiting hypomethylation following E2-treatment in young and middle-age, but not older animals, for select genes linked to hormone response signaling.

**Genes**	**CpG site YT-YC**	**CpG site MT-MC**
*Ahr*	Intergenic	Intergenic
*Andpro*	Intergenic	Intergenic
*Cyp4a2*	Intron	Intron
*Gpr22*	Intergenic	Intergenic
*Bcas1*	Intergenic	Intergenic
*Dpy19l4*	Intron	Intron
*Grin3a*	Intergenic	Intergenic
*Mbd2*	Intergenic	Intergenic
*Nalcn*	Intron	Intron
*Pth*	Intergenic	Intergenic

Several potential critical window genes are linked to the glutamate synapse. *Nedd4*, involved in synaptic plasticity, was responsive in young animals, but not in aged animals, for CpG, CGH, and CHH sites. The E2-sensitive glutamate receptor *Grm1* (Sarvari et al., 2017) exhibited E2-mediated hypomethylation for CHG and CHH sites in young and middle-age, but not in aged animals. Similarly, *Grm7* and *Grik2*, which are E2-responsive in the hippocampus during middle-age (Aenlle and Foster, 2010) exhibited decreased responsiveness for different sites (*Grm7* CpG sites and *Grik2* CHG sites) in aged animals. The NMDA receptor subunit, *Grin3a*, was responsive for CpG, CGH, and CHH sites in young and middle-age animals, but not in aged animals. The *Grin3a* subunit is thought to be involved in control of synapse maturation and is developmentally regulated in region CA1 of the hippocampus (Perez-Otano et al., 2016; Murillo et al., 2021). In adults, *Grin3a* may be linked to synaptic plasticity and the ability of estradiol to influence memory (Naderi et al., 2020). Finally, *Grin3a* may be of special interest with regards to the closing of the E2-responsive window, since the ability of E2 to enhance NMDA receptor function in region CA1 is a biomarker for the closing of the critical window (Vedder et al., 2014; Bean et al., 2015) and E2-responsiveness of *Grin3a* declines with advanced age (Aenlle and Foster, 2010). Finally, the *Grin3a* CpG methylation differences were observed for an intergenic region (Table 1). Whether this suggests changes in gene expression or chromatin structure is unclear.

Ovariectomy or estrogen replacement can modify age-related changes in DNA methylation (Levine et al., 2016; Stubbs et al., 2017). In other systems, a decrease in estrogen signaling promotes DNA methylation of E2-responsive genes (Leu et al., 2004; Stone et al., 2015; Levine et al., 2016). Indeed, methylation of the estrogen receptor rises with age and is associated with decreased receptor expression (Bean et al., 2014), consistent with the idea that the decline in estrogen signaling during aging may increase the vulnerability of E2-responsive genes to *de novo* DNA methylation (Bean et al., 2014; Barter and Foster, 2018). Increasing estrogen signaling activity may reverse some of these changes. However, due to the energy barrier of demethylating genes in preparation for transcription, expression may be delayed in older animals (Aenlle and Foster, 2010). Interestingly, the critical window for E2 effects on cognition and NMDA receptor function can be rejuvenated by increasing estrogen signaling, combining increased expression of estrogen receptor alpha (ERα) with E2-treatment (Bean et al., 2015).

Examining treatment effects within each age group provided conflicting evidence for E2 having a specific effect in reversing aging differences. E2-treatment resulted in clustering of similar GO terms and hypomethylation and hypermethylation of many of the same genes, within each age group. However, aged animals may be more sensitive to E2-induced changes in CpG and CHG sites, exhibiting more modified sites, particularly for islands, shores, and selves. In contrast, aged animals exhibited the fewest modifications of CHH genes associated with E2-treatment.

E2-treatment is most effective in enhancing cognition when the treatment is initiated perimenopausally. The decline in benefits with age suggests the closing of a therapeutic window. In the hippocampus, E2 has effects on physiology, transcriptional regulation, cell growth, and synaptic connectivity, which are opposite to that of aging and the ability of E2-treatment to modify the hippocampus declines with advanced age (Foster, 2005, 2012; Bean et al., 2014). The current study indicates that the mechanism for decreased E2-responsiveness with age includes a reduced ability for E2-induced hypomethylation of genes linked to hormone response pathways and regulation of synaptic function. This would imply that in younger animals, E2 may be acting through positive feedback mechanisms involving E2 signal activation and genes for proteins in the signaling cascade (Bean et al., 2014). A decrease in rapid E2-mediated signaling could limit subsequent activation of genomic mechanisms and contribute to delayed transcription of genes in the rapid signaling cascade (Aenlle and Foster, 2010). Furthermore, the decreased E2-responsiveness normally observed following E2 depletion during aging would be inhibited by E2-treatment to maintain E2 signaling (Daniel, 2013; Vedder et al., 2014) or signaling may be rejuvenated/recovered by enhancing E2 signaling in older animals (Bean et al., 2015).

## Data Availability Statement

The datasets presented in this study can be found in online repositories. The names of the repository/repositories and accession number(s) can be found in the article/Supplementary Material.

## Ethics Statement

The animal study was reviewed and approved by the Institutional Animal Care and Use Committee (IACUC) at the University of Florida.

## Author Contributions

PS performed the experiments, analyzed the data, constructed illustrations, and wrote the manuscript. ARa performed the experiments and analyzed the data. AK designed the experiments, performed the experiments, and wrote the manuscript. ARi and JB analyzed the data. TF designed the experiments, analyzed the data, constructed illustrations, and wrote the manuscript. All authors contributed to the article and approved the submitted version.

## Conflict of Interest

The authors declare that the research was conducted in the absence of any commercial or financial relationships that could be construed as a potential conflict of interest.

## Publisher’s Note

All claims expressed in this article are solely those of the authors and do not necessarily represent those of their affiliated organizations, or those of the publisher, the editors and the reviewers. Any product that may be evaluated in this article, or claim that may be made by its manufacturer, is not guaranteed or endorsed by the publisher.
